# Teaching Self-efficacy and Teaching Methods in the Aquatic Environment

**DOI:** 10.12688/f1000research.150535.1

**Published:** 2024-11-14

**Authors:** Rita Fonseca-Pinto, Juan Antonio Moreno-Murcia

**Affiliations:** 1 Universidad Miguel Hernandez de Elche, Elche, Valencian Community, Spain

**Keywords:** methods, aquatic literacy, teaching, self-efficacy

## Abstract

**Background:**

Studies have shown that perceived self-efficacy can influence teachers’ emotional state, thoughts and behaviours, and students’ learning. It’s also an important referential of professional satisfaction. In turn, teaching methodologies influence motor learning, as well as psychological, cognitive and social learning, with different impacts on human development and learning retention, levels of intrinsic motivation and continuity of practice in order to support a healthy lifestyle. Research on aquatic educators and teaching methodologies is scarce and at the same time necessary according to the view that aquatic literacy is an integral part of physical literacy and the only possibility of being more able to interact with this environment.

**Methods:**

In this study we used an online questionnaire, aimed at aquatic professionals, which was answered voluntarily and anonymously to measure the prevalence of the use of different teaching methodologies, the comprehensive aquatic method and the perception of teacher self-efficacy. It has been deposited and can be consulted at https://doi.org/10.6084/m9.figshare.27316242.v1.

**Results:**

All methods can generate a feeling of self-efficacy in teachers despite having different results, with the methodologies that involve students more actively (cognitivist and constructivist) being those that generate a greater feeling of self-efficacy in teachers. MAC is a method that is more closely related to methodologies focused on active student participation and, consequently, it is a method that generates a high perception of self-efficacy in teachers.

**Conclusions:**

Levels of self-efficacy influence professional satisfaction, teacher physical, mental and emotional health, as well as student learning. Is recommended that aquatic professionals give prevalence to the cognitivist and constructivist teaching methodologies being MAC a privileged methodological approach for promoting active lifestyle habits throughout life.

## Introduction

Considering aquatic literacy as an integral part of physical literacy ( Albarracín & Moreno-Murcia, 2018), allows to increase the vision on the contents and impact of school physical education. The development of aquatic literacy, as part of a larger concept called water competence, in the school-aged population can contribute to the reduction of physical inactivity and is certainly determinant for the prevention of drowning not only at school age but also in the adult future. In order to reach as many children and youth as possible, it is necessary to make the practice accessible to all, and only the school has this inclusive opportunity. But it is not enough the content that can be offered to the students, it is important to value how this content is shared considering the benefits for the teacher, for the student and the retention not only of the learning but more than anything else of the enjoyment of an active lifestyle, which finds in the aquatic activities one more way to stay active in the long term. In this sense, the study of teacher self-efficacy, considered as a key element for professional satisfaction, motivation ( Ortan et al., 2021) and emotional self-regulation of the professional ( Shahzad & Naureen, 2017), has been considered a priority in the field of the specificity of the professionals responsible for the aquatic education of school children. According to Bandura’s (1977, 1997) theory, self-efficacy refers to people’s beliefs about their abilities to successfully carry out the actions necessary to complete a specific task. In turn, and according to the view of, Ainley & Carstens (2018) define self-efficacy as “the beliefs that teachers have about their ability to implement certain teaching behaviours that influence students’ educational outcomes, such as achievement, interest and motivation”. These beliefs are influenced by experience and, more specifically in education, are reflected in teachers’ beliefs that they can perform behaviours that produce results or outcomes. These types of personal beliefs tend to be rigid ( Ortan et al., 2021). So, from a theoretical perspective, teacher self-efficacy is a multi-faceted affective construct ( Zakariya 2020).

People with a high sense of self-efficacy tend to see themselves in a successful situation, which is a positive incentive in their performance ( Schwarzer et al., 2000), are more confident and have greater pro-activity in task solving ( Peng & Mao, 2015). In contrast, people with a low sense of self-efficacy are more prone to depression, anxiety ( Bandura, 1997) and to have more problems with student discipline and behaviour ( Ortan et al., 2021). Perceived teacher self-efficacy is an essential element that can influence teachers’ thoughts, behaviours and emotions ( Bandura, 1997; Pendergast et al., 2011; Poulou et al., 2019), the receptiveness to experiment new methods in an attempt to meet the needs of the learners ( Shahzad & Naureen, 2017), as well as student learning ( Dellinger et al., 2008). However, most studies on teacher self-efficacy seem to focus predominantly on job satisfaction ( Zakariya, 2020).

For several decades, attempts have been made to discover those teaching characteristics that best demonstrate high effectiveness in professional work ( Korthagen, 2004; Koster et al., 2005), in order to configure an effective teaching style and thus make it possible to link teaching variables with self-efficacy ( Avalos, 2011).

Tschannen-Mora & Woolfolk Hoy (2001) review all the sources in which the construct of teacher efficacy appears, concluding that teacher self-efficacy is not only related to student outcomes in terms of performance, motivation and self-efficacy, but is also to some extend linked to the behaviours that students display in the classroom as a result of the teacher’s methodological intervention. In this sense, relating information on the characteristics of teaching methodology (from more behaviourist to more constructivist perspectives) and teacher self-efficacy could help to determine which practices lead to greater benefits.

### Teaching methodologies

A diverse range of teaching methodologies exists in educational contexts, often categorized as autonomy-supportive or controlling. The teacher’s approach significantly influences student learning outcomes, determining whether students succeed or struggle academically ( Shahzad & Naureen, 2017; Woolfolk, 1998). Research indicates that teaching methodologies in sports not only affect technical, tactical, and physical skills but also psychological and social dimensions ( Emmanouel et al., 1992). For instance, in aquatic skill instruction, the teacher’s influence extends beyond motor skills, impacting levels of intrinsic motivation.

Healthy variability in teaching ( Mosston & Ashworth, 2002) offers multiple, non-exclusive interaction options between teacher and student, emphasizing the importance of decision-making skills ( Hein et al., 2015). One method is the reproductive methodology, characterized by a strictly teacher-directed approach where students merely replicate tasks. This behaviorist model relies on environmental reinforcement and leads to rote, repetitive learning based on stimulus-response dynamics ( Hein et al., 2012).

In contrast, the productive, learner-centered methodology ( Bechter et al., 2019) focuses on knowledge acquisition through creating mental structures and understanding learning processes ( Dyson et al., 2004). Here, learners actively engage in problem-solving, evaluating alternatives based on their knowledge ( Hein et al., 2012).

The constructivist methodology shifts the focus to students, allowing them to set learning objectives, select content, and choose assessment methods ( Barker et al., 2014; Kirk & Macdonald, 1998). This approach fosters active participation, enabling students to solve problems with teacher support, thereby constructing their own understanding of the subject matter ( Nuñez & Oliver, 2020; Goodyear & Dudley, 2015).

A fourth approach, known as laissez-faire, involves minimal teacher intervention, where the teacher only organizes equipment and manages time and space without engaging with students during activities ( Hein et al., 2012).

The evolution of pedagogy reflects a shift from instructional to constructivist methodologies, demonstrating that student-centered approaches yield significant benefits ( Barker et al., 2014; Bechter et al., 2019; Walseth et al., 2018). More passive teaching methods, characterized by authoritative directives, can hinder athletes’ autonomy, leading to anxiety, boredom, and indiscipline ( Reeve & Tseng, 2011). Such methods often focus on isolated, non-contextualized tasks that fail to engage students as active learners, diminishing their learning outcomes ( Blomquist et al., 2001).

Conversely, active involvement methods encourage students to become creative, social learners who construct their own knowledge and identify areas for improvement ( Dyson et al., 2004). These approaches emphasize the simultaneous development of technical skills, tactical understanding, and decision-making through real-game situations and problem-solving, exemplified by Teaching Games for Understanding (TGfU) and the Comprehensive Aquatic Method (MAC) ( Moreno-Murcia & Ruiz, 2019).

Introduced by Moreno and Gutiérrez in 1998, the MAC is a constructivist approach that centers on student interests and needs, promoting autonomy and interaction. It employs play as a medium, with the educator fostering a positive, varied learning environment ( Moreno-Murcia & González, 2022). This methodology encourages problem-solving and exploration in aquatic environments, developing knowledge, skills, and attitudes that contribute to student autonomy and personal growth ( De Paula & Moreno-Murcia, 2018). Such models facilitate opportunities for all students to solve problems, make decisions, and demonstrate leadership ( Metzler, 2011), equipping them with essential competencies for various life stages and contexts.

Utilizing cognitive and constructivist methods in sport education can enhance information retention and enjoyment ( Batez et al., 2021). This focus is crucial for increasing youth engagement in regular physical activity, especially considering that many students would opt out of physical education if given the choice, with over 50% of 10th and 11th graders expressing disinterest in the current curriculum ( Ha, Johns & Shiu, 2003). Addressing challenges in identifying relevant PE content, actively engaging students, and building self-efficacy among educators and learners is essential for fostering health and social well-being through physical activity, with aquatic activities playing a critical role in drowning prevention.

## Teaching self-efficacy and teaching methods

Self-efficacy is a construct that has been analysed in the field of education in general and in specific contexts, such as school physical education and physical-sport activity ( Balaguer et al., 1995) and is one of the most widely used theories in sport teaching and motor performance ( Feltz, 1995). An extensive number of studies have accumulated over decades on the basis of Bandura’s (1986) social cognitive theory, especially in relation to people’s beliefs about their abilities, i.e., about self-efficacy evaluations and achievement behaviours in sport ( Beauchamp et al., 2019). For example, the study by Moritz et al. (2000) found that self-efficacy is a consistent correlate of performance in sport. In this sense, the nature and direction of the efficacy-performance relationships differ depending on whether intrapersonal or interpersonal effects are considered ( Beattie et al., 2016; Vancouver, 2018). That is, while athletes who display high self-efficacy beliefs in relation to others may be more likely to show better outcomes and performance, the nature of the efficacy-performance relationship may differ markedly when intra-individual effects are examined.

Some studies ( Vancouver & Purl, 2017) have shown that when task-related information and feedback are ambiguous, within-person effects tend to be negative, but when such information and feedback are unambiguous, within-person effects tend to be positive. When participants are provided with performance feedback, higher levels of self-efficacy are related to better subsequent performance, and when feedback is not available, the self-efficacy-performance relationship is negative ( Beattie et al., 2016). This suggests that the way of teaching (teaching method) used by the teacher can help participants to achieve their present and subsequent achievements. The methodology implemented by the teacher can help to facilitate the construction of particular self-efficacy beliefs, as long as individuals have sufficient information on which to base their judgements of ability ( Beauchamp et al., 2023) and in continuity self-efficacy allows the teacher to optimise and adjust the teaching method, but also the teacher-student, teacher-family interactions, cooperation between the elements of the educational team, decision-making and the atmosphere in the educational environment ( Chacón, 2005; Ortan et al., 2021).

Therefore, self-efficacy is based on a person’s beliefs about his or her own ability to plan, organise and execute the actions necessary to reach a certain achievement ( Bandura, 1997). Specifically, in teaching action, this is an intrapersonal motivational variable that helps to increase students’ persistence, commitment and enthusiasm for practices ( Skaalvik & Skaalvik, 2007). Beliefs about teaching effectiveness are constructed on the basis of two elements of the teaching-learning process: the task and the context ( Chao et al., 2017). In this sense, the effective teacher should master different teaching methodologies and know how to adjust them to the needs of each context.

Evidence indicates that, in general, teachers with higher self-efficacy are more likely to manage the classroom effectively ( Tschannen-Moran & Woolfolk Hoy, 2007), to use more constructivist and higher quality instruction ( Holzberger et al., 2013; Suprayogi et al., 2017), developing challenging approaches ( Deemer, 2004), using creativity-focused instruction and comprehensive and meaningful learning ( Deemer, 2004), encouraging learner autonomy and keeping learners motivated on task ( Chao et al., 2017; Miller et al., 2017). Also, high self-efficacy values are associated with professionals who are more passionate about their teaching mission ( Fernet et al., 2014), with greater control over their emotions, and even have less tendency to show anger or rage in front of their students’ behaviour ( Shahzad & Naureen, 2017). It is very clear that the perception of teacher self-efficacy influences their professional practice with determining results in the learning processes of students ( Pan, 2014) in the generality of educational processes where physical education is an important part, as it has a direct relationship with the promotion of active lifestyle habits and can influence the rate of personal aquatic competence from childhood through the practice of physical activity in the aquatic environment guided by professionals committed to their mission.

### The current study

In order to analyse the methodologies chosen most frequently by aquatic educators, it is important to know their self-efficacy belief system (what I feel confident about doing, what I think the students’ role should be in class, how to act so that they learn, how to act to control/avoid indiscipline, etc.), as this is the main basis for the professional’s decision making when planning and directing the activity. There is a tendency to choose the activities in which one feels more confident to the detriment of those in which one feels less competent.

Teacher self-efficacy is a critical factor that variably influences (positively and negatively) student performance, being more significant than class size, socio-economic status or the starting level with which the student arrives ( Darling-Hammond & Youngs, 2002; Hindman & Stronge, 2009; Klassen & Tze, 2014; Staiger & Rockoff, 2010). There are numerous studies on self-efficacy in the context of Physical Education ( Curran & Standage, 2017; De Meyer et al., 2016; Leo et al., 2020), however, there are few studies that relate teachers’ self-efficacy beliefs in practices and how they actually use these practices in their classroom ( Poulou et al., 2019). Furthermore, we have not found research that verified the relationship between teaching methodologies and teacher self-efficacy in teaching in the aquatic environment.

For this reason, we consider answering the following questions related to the aquatic professionals’ belief system on teaching methodologies and self-efficacy from the teaching perspective: What is the aquatic professional’s perception of self-efficacy? What teaching methodologies does the most effective professional use?

The main objective of this study was to analyse the relationship between teaching methodologies, the comprehensive aquatic method and teaching self-efficacy, as well as to check which type of teaching methodology (cognitive, constructivist, behaviourist or laissez-faire) predicted the comprehensive aquatic method. Considering the results of the previously mentioned research, it is expected to find a predictive model where methodologies that actively involve students will positively predict the comprehensive aquatic method in aquatic educators. It is also expected that teacher self-efficacy will be predicted by the most learner-involving methodologies (constructive, cognitive and MAC).

## Methods

### Participants

A total of 607 aquatic educators (305 males and 302 females) aged between 18 and 61 years (M = 36.58, SD = 13.02) participated in this research and during february and april from 2024 the data analysis was done. The selection criteria for the sample were that they were at least 18 years old, with at least 6 months of teaching experience in the aquatic environment and that they were in possession of a qualification. By age, they were classified into three groups: under 25 years old (n = 162), between 25 and 40 years old (n = 197) and over 40 years old (n = 248). The educators’ training was as follows: graduates in Physical Activity and Sport Sciences (n = 147), Physical Education Teachers (n = 285), Sports Technicians (n = 102) and other qualifications (n = 73). According to the years of experience of the aquatic educators, they had the following values: less than 1 year (n = 75), between 1-5 years (n = 75), between 6-10 years (n = 277) and more than 10 years (n = 92). The aquatic educators who participated in the study were teaching students aged 0-6 years (n = 126), 7-12 years (n = 117), 13-18 years (n = 41), over 18 years (n = 36) and all ages (n = 287).

### Measures

This study used three questionnaires from different authors and content that together address the issues in question, and their use has been possible with permission obtained from all authors of wich type of tool.

*Comprehensive aquatic method (MAC).* The scale validated by Castañón-Rubio et al. (2022) was used with permission obtained from the authors to use these tool, to measure the use of the comprehensive aquatic method in aquatic educators. This scale consisted of 26 items divided into 3 subscales as follows: a) way of teaching, consisting of 10 items (e.g. “In each class I evaluate the objectives”); b) game, consisting of 9 items (e.g. “I adapt the games taking into account the resource and characteristics of the aquatic installation”); c) the way of teaching, consisting of 10 items (e.g. “I offer possibilities of choice (groupings, materials, etc.) to participants.”). Responses were recorded on a Likert scale from 1 (strongly disagree) to 5 (strongly agree). The internal consistency obtained for each of the dimensions was .93, .88, .87, respectively, and .91 for the global MAC, considering the three dimensions as one.

*Teaching methodologies.* In order to analyse the teachers’ perception of the use of their methodology, the adaptation of the Teaching-Learning Methods Scale in Physical Education (TLMS-PE; Leo et al., 2020) was used with permission obtained from the authors. This questionnaire is composed of the introductory sentence (e.g., “In my classes…”), followed by 19 items organised in five dimensions: 5 items to assess the reproductive methodology (e.g., “I determine at all times the activity they should practice”), 5 items to assess productive methodology (e.g., “I make them look for and evaluate different answers and solutions to the tasks I set”), 6 items to assess the constructivist methodology (e.g., “I give participation to the students so that they can decide what they want to learn”) and 3 items to assess letting them do during the classes (e.g., “I spend time intervening at the beginning and end of the class distributing the material for them to play with”). The response format used is Likert-type with a 5-point response range from 1 (strongly disagree) to 5 (strongly agree). Reliability analysis showed how the items belonging to these variables were reliable for constructivist (.78), productive (.83), reproductive (.74) and laissez-faire (.70) methodology.

*Teacher self-efficacy.* In order to assess teaching self-efficacy, was used an adapted version of the Teachers’ Sense of Efficacy Scale by Tschannen-Moran and Woolfolk (2001) with permission obtained from the authors to use these tool. The 12 items of this scale measure perceived teacher self-efficacy in three dimensions: a) perceived efficacy in optimising one’s own instruction (4 items); b) perceived efficacy in classroom management (4 items) and c) efficacy in involving students in learning (4 items). The reliability of the total scale was .92, while by dimensions it was .81, .86 and .81, respectively.

### Procedures

The present research has the approval of the relevant University Human Research Committee with the reference DCD.JMM.01.22, as well as all the scales used have obtained the respective permissions from their original authors. In order to carry out the data collection, different aquatic centres were contacted to request their collaboration in the study. The research team subsequently administered the questionnaire in an online format, which was answered voluntarily and anonymously. The researchers were also available to answer any questions that arose during the process of completing the questionnaire. The questionnaire took between 15-20 minutes to complete.

### Data analysis

Descriptive statistics (means and standard deviations) and bivariate correlations were calculated for all the variables under study. Cronbach’s alpha coefficient was used to determine the internal consistency of each of the dimensions. Also, the predictive power of the teaching methodologies on Comprehensive Aquatic Method (MAC) was tested by means of a stepwise multiple linear regression analysis. A significance level of p < .05 was used for the statistics obtained in the different analyses, which were carried out using the R Project for statics computing ( R Core Team, 2023).

## Results

### Descriptive statistics and correlations of all variables

The most valued variable in the MAC was the autonomy-supportive teaching style, followed by the teaching style and play. In relation to teaching self-efficacy, the most important dimension was instruction, followed by strategies for involving students in learning and classroom management. Regarding teaching methodologies, cognitive methodologies were the most valued, followed by behavioural, constructivist and laissez-faire ( Table 1). Correlation analysis revealed the existence of a positive and significant relationship between all the dimensions (p < .01), except between the game and behaviourist dimensions (p < .01), which was negative and significant. No significant correlations were found between the dimensions way of teaching and interpersonal style and behavioural methodologies and letting do, and between play and letting do (p > .05).

**Table 1.** Descriptive statistics and correlation analysis of all variables.

	*M*	*DT*	1	2	3	4	5	6	7	8	9	10
1. Way of teaching (MAC)	3.16	.48	-	.60 ^**^	.60 ^**^	.17 ^**^	.23 ^**^	.21 ^**^	.10 ^**^	- .01	.18 ^**^	- .03
2. Game (MAC)	3.12	.53	-	-	.67 ^**^	.20 ^**^	.22 ^**^	.22 ^**^	.25 ^**^	- .08 ^*^	.30 ^**^	.07
3. Interpersonal style (MAC)	3.21	.47	-	-	-	.22 ^**^	.23 ^**^	.22 ^**^	.25 ^**^	- .04	.27 ^**^	- .00
4. Commitment (Self-efficacy)	8.01	.98	-	-	-	-	.77 ^**^	.75 ^**^	.29 ^**^	.19 ^**^	.40 ^**^	.13 ^**^
5. Teaching (Self-efficacy)	7.92	1.04	-	-	-	-	-	.75 ^**^	.27 ^**^	.22 ^**^	.43 ^**^	.12 ^**^
6. Management (Self-efficacy)	7.87	1.00	-	-	-	-	-	-	.18 ^**^	.22 ^**^	.34 ^**^	.12 ^**^
7. Constructivist (Methodology)	3.62	.77	-	-	-	-	-	-	-	.18 ^**^	.67 ^**^	.57 ^**^
8. Behaviourist (Methodology)	3.79	.69	-	-	-	-	-	-	-	-	.30 ^**^	.37 ^**^
9. Cognitivist (Methodology)	4.02	.69	-	-	-	-	-	-	-	-	-	.42 ^**^
10. Laissez-faire (Methodology)	3.11	.94	-	-	-	-	-	-	-	-	-	-

### Linear regression analysis

A stepwise linear regression analysis was undertaken to find out how the different methodologies used predicted the MAC. According to the last step in the linear regression analysis, the different methodologies used predicted MAC with an explained variance of 12% ( Table 2). The methodologies that positively predicted MAC were cognitive (ß = .29, p < .01) and constructivist (ß = .13, p < .05), while behaviourist (ß = -.12, p < .05) and let-do (ß = -.14, p < .05) methodologies predicted it negatively.

**Table 2.** Linear regression analysis of prediction of the MAC across teaching methodologies.

	*B*	*SEB*	*b*	*R* ^2^
Teaching methodologies	2.63	.12		.12 **
Constructivist	.07	.03	.13 *	
Behaviourist	- .07	.02	- .12 *	
Cognitivist	.18	.03	.29 **	
Laissez-faire	- .06	.02	- .14 *	

* *p <* .05.

** *p* < .01.

A stepwise linear regression analysis was conducted to test which variables (teaching methodologies and MAC) best explained teaching self-efficacy. According to the last step in the linear regression analysis, cognitive (ß = .35, p < .01), behavioural (ß = .16, p < .01), and MAC (ß = .18, p < .01) methodologies positively predicted teaching self-efficacy with a total explained variance of 23% ( Table 3).

**Table 3.** Linear regression analysis of prediction of teaching self-efficacy across different teaching methodologies and the MAC.

	*B*	*SEB*	*b*	*R* ^2^
	4.20	.32		.23 **
Constructive methodology	.01	.06	.01 **	
Behavioural methodology	.21	.05	.16	
Cognitive methodology	.47	.06	.35 **	
Laissez-faire methodology	-.07	.04	-.07	
MAC	.38	.08	.18 **	

* *p <* .05.

** *p* < .01.

## Discussion

Full confidence in one’s own efficacy is needed in order to begin and sustain the effort to achieve success ( Mone et al., 1995). Therefore, teacher self-efficacy may be essential for classroom success, as evidence ( Miller et al., 2017) shows its relationship with teaching quality, student achievement and job satisfaction ( Toropova et al., 2021). Most of the studies that analyse the relationships between different methodologies and general self-efficacy have focused on students and other contexts, so this research is a first approach to the study of these variables jointly on teachers in the aquatic education environment. With this in mind, the main objective of the study was to analyse the relationship between teaching methods, the MAC, and the teaching self-efficacy perceived by aquatic educators. In general terms, the results confirm our initial hypotheses, the MAC is more related to methodologies that actively involve students and is positively predicted by cognitive and constructivist methodologies. Furthermore, teacher self-efficacy is predicted by the methodologies that most actively involve the learner (constructivist, cognitive and MAC). These references are in line with Guskey’s (1988) study where the more effective teachers showed greater confidence and diversity of teaching strategies with creative and interesting pedagogical approaches in the classroom, contrary to the tendency of the less effective teachers to choose more traditional methods. The assessment of the development of water competence in physical education curricula can be considered as an innovative pedagogical proposal that more effective teachers are more inclined to consider and which we believe will have a positive effect on the future water competence index of society in general. Achieving this will depend on the high degree of flexibility needed to generate and adapt to new scenarios, where both teachers and pupils benefit ( Shahzad & Naureen, 2017; Pan, 2014). This is essential, recognising the importance of benefiting pupils and teachers in the development and promotion of a more active and consequently healthier lifestyle.

In our study, all methods (constructivist, behavioural, cognitive, laissez-faire and MAC) have shown a positive relationship with the perception of self-efficacy in aquatic professionals. In general, science has identified that the use of more learner-centred methodologies generate a greater sense of teacher self-efficacy than the others, but with all of them it is possible to generate a sense of self-efficacy, although at different levels. Consequently, lower levels of self-efficacy are predictors of teacher burnout and stress ( Dicke et al., 2014; Zhu et al., 2018) and lower student achievement ( Sharma et al., 2012). Although teachers do not perceive different methods to be equally effective in all teaching situations (it depends on the task and context), it is the cognitive and constructivist methodologies that generate the most self-efficacy perceptions ( Chao et al., 2017; Miller et al., 2017; Suprayogi et al., 2017). A constructivist view sees learning as a process of adapting and fitting into an ever-changing world and the aquatic environment, in its broadest expression, something is learned beyond what is practised in a pool or controlled environment. Interaction with the aquatic environment occurs through neuroception, perception, motor action and senses, so we build through the perception that the body gets from the experience ( Light, 2008). In this sense and analysing the strong correlation of MAC with these methodologies focused on the students (for their autonomy and transfer of competences) and their needs, it is possible to affirm that MAC, due to its proximity to cognitivist and constructivist methodologies, when used, teachers will feel more self-effective and well succeeded in their role of contributing to the development of water competences and promoting students’ intrinsic motivation for physical activity through water activities.

A dualistic view of the human being as mind and body (separation of self and action) is still common from a social point of view, and in this sense, following the approaches of behaviourist methodologies, the emphasis is still exclusively on the use of feedback and reward systems to alter and modify behaviour. But as Light (2008) points out, in a holistic view, the body and sensation are equally important in learning. So for lifelong use, the choice of the methodology to be used for better learning and retention will depend on the content to be covered, the age of the learner, their degree of autonomy and the effects expected.

There are several reasons that may explain the use of certain teaching methodologies, such as, for example, the development of motor skills and the improvement of sport-specific skills ( Dudley et al. 2011; Hardman, 2008). There is often a tendency to use more behavioural teaching styles designed to help students develop correct technique while providing maximum practice time ( Chatoupis & Vagenas, 2017; Goldberger, 1984, 1992), but teaching motivation must go beyond the intention of developing motor competence in participants or teaching competitive sporting activities ( Chatoupis, 2018). It is not only about influencing children and young people in a certain content or set of contents focused on how to do, but that this perception of teaching self-efficacy can extrapolate its scope to the development of personal and social skills (areas of to know and to be, individually and in groups), through active learning on the part of the students, involved in an attractive way in the process ( Ledertoug and Paarup, 2021).

We interpret that the use of behaviourist models leading to a perception of teacher self-efficacy may be due to: (a) the need for the aquatic educator to guarantee the safety of the students, as the aquatic environment has a possible risk of drowning built into it; (b) by tradition, as they have historically learnt this in their sports training centres and have not had the possibility of learning about other more efficient models; (c) by the pattern followed by technical models centred on sporting skill, which emphasise knowing how to do and reproduce, where deciding or thinking is not a necessity.

In the light of the contributions of this study, it is important to emphasise the role played by teachers in the design of their classes. In general, teachers feel more effective when their methodologies involve active learning, making students the protagonists of the process, giving them responsibility and autonomy, encouraging their creativity instead of meaningless memorisation, and teaching them to think in order to successfully solve the problems they face. But according to the study by Lubans et al. (2017) they are not reaching their potential and in order for the sessions to have a greater effect on the level of physical activity and physical literacy of young people it is essential to consider the quality of the tasks and for that it is important to design tasks with methodologies that actively involve the learner, in order to get the learner to focus more on mastery. This would be possible by giving more importance to aspects of self-improvement and learning, contributing to a perception of personal progress in a more effective way, feeling autonomous, competent and assuming a modifiable belief in their ability. These factors will allow them to overcome intrinsically motivated tasks, resulting in a positive psychological balance that can generate greater enjoyment and less boredom on the part of the students, as the enjoyment experienced in classes can be an important variable for greater learning. Extending the traditional to do approach into to know and to be may be one of the possible and decisive strategies, since in order to learn the brain needs to be excited and validated as meaningful. In terms of interaction with the aquatic environment, the development of this kind of knowledge is fundamental to make transfers of knowledge from educational environments to real environments more probable. The role of aquatic education as an integral part of school physical education can clearly make a difference, as it would imply learning by professionals who are aware, committed and competent about their mission.

Despite the strengths of the study, there are a number of limitations to be taken into account when interpreting the results. Firstly, the teaching-learning methodologies are assessed from the point of view of the teacher and not the students, which may not reflect the true methodology used. Secondly, being a correlational study, the results do not indicate the existence of a cause-effect relationship between the variables considered. In relation to future prospects, it would be interesting to analyse the effect of the different methodologies in an intervention and to check whether the relationship presented in this study is confirmed in practice. In addition, it could be relevant to establish experimental studies with different groups with different methodologies and to analyse the benefits or consequences of each of them. Given the potential of self-efficacy beliefs to both enhance and impair performance, future research on self-efficacy could assess associations within individuals by analysing high quality feedback or information related to task performance and the methodology that has been used to generate goal achievement.

The intention professional who teaches aquatic education, independent of the educational setting, is that their students learn as much as possible regardless of the methodology they use. Their decisions are based on personal beliefs built up over time and influenced by some learning theories, which are rarely questioned ( Rink, 2001). As the study by Toropova et al. (2021) indicates, this perception of self-efficacy varies with years of experience, being lower in less experienced professionals and declining in pre-retirement teachers.

This has been building cultural behaviours in the teaching methodology of the teacher in the aquatic context, which we can partly see reflected in this first study. In the search for true learning, evidence is needed in this regard, and it is challenging for trainees to implement more constructivist perspectives ( Rovegno, 1998), as the starting point is to consciously question beliefs and relate them to the assumptions of learning ( Davis & Sumara, 2003). It is necessary to understand learning theory and be aware of personal beliefs about the assumptions of what it means to be “‘water competent’” ( Fonseca-Pinto & Moreno-Murcia, 2023). In this way, aquatic educators’ decisions should be adapted to the needs of the learner and the context.

## Pedagogical implications

One of the main approaches to combat physical inactivity emphasises the need to improve the quality of activities, making them highly active and engaging ( Hills et al., 2015). To encourage lifelong physical activity, counteracting current levels of inactivity, it is crucial to raise the levels of intrinsic motivation of practitioners. A multi-sectoral and multi-systemic approach is recommended, where both in-school and out-of-school programmes promote physical activity even outside the school environment ( Lubans et al., 2017). However, in reality, the full potential of these programmes has not been fully realised. What are the possible contributions of this study?

Aware that there is still much to explore, given that the evidence is limited from both teacher and student perspectives, and that there is a two-way relationship that should not be neglected, this study presents cross-cutting aspects to other published studies, reinforcing the idea that these results can have direct implications for the teaching function, either in the field of school physical education, sports or extracurricular physical activities.

In this study, a relationship is observed between teaching methodologies and the level of self-efficacy perceived by the teacher. Cognitive and constructivist methodologies are the ones that best explain teacher self-efficacy. Although it is possible that teachers perceive themselves to be self-efficacious with all teaching methodologies, it is noteworthy that the values are higher when predominantly cognitivist and constructivist methodologies are chosen. There is a strong relationship between teacher self-efficacy, student self-efficacy, learning outcomes, job satisfaction and retention in the teaching career, indicating that teachers who wish to feel more effective should predominantly opt for these practices. However, it is important to note that the choice of teaching methodology is influenced by context, timing and learner needs.

Higher teacher self-efficacy and the flexibility to manage the class with different methodologies and strategies allow for active student participation during the learning process, with positive effects on student motivation and learning climate ( Pan, 2014). We recommend that future studies assess levels of personal motivation, enjoyment of physical activity in general or in specific sports, the influence of student motivation in the subject of physical education, and the teacher behaviours that most influence students’ intrinsic motivation to consider an active life by practising aquatic activities throughout life.

According to the MAC methodology, the most highly valued variable was the teaching style of supporting autonomy and play. These teaching strategies are directly related to cognitivist and constructivist methodologies, also associated with higher values of perceived teacher self-efficacy. In summary, if the teacher seeks a greater positive impact on their students from an ecological and holistic perspective of pedagogy, and wishes to feel more self-effective, they should choose MAC as a methodology that is present and active in their teaching role.

There is no infallible method, but there is the ability to adjust methodologies to the circumstances. The MAC, as an example of a constructivist methodology, together with cognitivist and even, in some cases, behaviourist methodologies, can fulfil the function of the learning moment considering the student as the protagonist. To achieve this, lessons should be oriented with an educational and competence development objective, considering equally important to know, to do, know and to be Ledertoug and Paarup (2021), with an emphasis on aquatic activities due to the effect they can have on drowning prevention. It is crucial that the practitioner, whether in the context of school, sports or extracurricular activities, considers these indications because of the importance of decision-making and knowledge transfer to other settings. This transfer perspective also considers the lifelong practice of physical activity, which is essential to maintain high levels of personal water competence when aquatic activities are part of a more active lifestyle.

In addition to what is considered in this study and based on the evidence presented, we highlight that the perception of self-efficacy varies according to years of experience ( Toropova et al., 2021). It is important to value specialised and highly complex continuous training to deepen knowledge about teaching and learning in the educational context ( Catalano et al., 2020). The specificity of physical education, and in particular the protective role of water competence in drowning prevention, underlines the importance of this development in teachers’ professional and personal development.

Given that teacher self-efficacy is key to both professional motivation and emotional self-regulation of teachers, high levels of self-efficacy will positively influence the reduction of the likelihood of burnout processes ( Ortan et al., 2021), thus preserving the physical, emotional and mental health of the professional, and directly benefiting the learner and learning.

## Conclusions

Levels of self-efficacy influence professional satisfaction, teacher physical, mental and emotional health, as well as student learning. Because of the positive and transversal impact that teaching intervention in school physical education classes or as a physical activity (extracurricular practice) can have on the personal water competence index, it is important to ensure high levels of teacher self-efficacy in aquatic education. All methods can generate a feeling of self-efficacy in teachers despite having different results, with the methodologies that involve students more actively (cognitivist and constructivist) being those that generate a greater feeling of self-efficacy in teachers, so is recommended that professional give preference to these methodologies. MAC is a method that is more closely related to methodologies focused on active student participation and, consequently, it is a method that generates a high perception of self-efficacy in teachers. And since the personal water competence index is socially important, we recommend, based on the data obtained, that MAC be considered a privileged methodological approach for promoting active lifestyle habits throughout life.

### Ethical approval and consent

This study is part of a doctoral thesis organised into three parts related to aquatic competence and teaching methodologies. Specifically, this study addresses the issue of teaching methodologies in aquatic activities and the perception of self-efficacy by aquatic professionals. The University’s Ethics Committee has had access to the study project as a whole, which has been approved by the Miguel Hernández Ethical Committee with the DCD.JMM.01.22 code on 6 of February of 2024. All the participants in this study are adults and before started to answer the survey provided a written informed consent by agreeing to participate saying yes to the first question and respond to the complete questionnaire.

## Data Availability

Fonseca-Pinto, R., & Moreno-Murcia, J.A. (2024b). *Teaching self-efficacy and teaching methods in the aquatic environment.* [Dataset]. Figshare. Online. http://doi.org/10.6084/m9.figshare.27310473 The project contains the following underlying data:
• Data.xlsx. (anonymised answers to questionnaires) Data is available under the terms of the Creative Commons Attribution 4.0 International license (CC-BY 4.0). Fonseca-Pinto, R., & Moreno-Murcia, J. A. (2024a). *(Survey) Teaching Methods in Aquatic Education.* [Dataset]. Figshare. Online. https://doi.org/10.6084/m9.figshare.27316242.v1 The project contains the following extended data:
•
*Teaching methods in aquatic education* (survey questionnaire). Data is available under the terms of the Creative Commons Attribution 4.0 International license (CC-BY 4.0).
