# Supplementary material for: Teaching Self-efficacy and Teaching Methods in the Aquatic Environment
Source: F1000Res. 2025 Mar 4;13:1363. Originally published 2024 Nov 14. [Version 2] doi: 10.12688/f1000research.150535.2 (PMC11904400; doi:10.12688/f1000research.150535.2)
Supplement: Peer review history

# Reviewer report: Reviewer response for version 2

**DOI:** 10.5256/f1000research.178323.r369463

**Published:** 2025-03-12

**Authors:** Ana Rita Matias

**Affiliations:** 1 University of Évora, Evora, Portugal

The authors answered the questions I mentioned satisfactorily. For this reason, I have no further comments to make

Is the work clearly and accurately presented and does it cite the current literature?

Partly

If applicable, is the statistical analysis and its interpretation appropriate?

Partly

Are all the source data underlying the results available to ensure full reproducibility?

Partly

Is the study design appropriate and is the work technically sound?

Partly

Are the conclusions drawn adequately supported by the results?

Yes

Are sufficient details of methods and analysis provided to allow replication by others?

Partly

Reviewer Expertise:

Aquatic therapy; child development; learning development

I confirm that I have read this submission and believe that I have an appropriate level of expertise to confirm that it is of an acceptable scientific standard.

# Reviewer report: Reviewer response for version 1

**DOI:** 10.5256/f1000research.165114.r341215

**Published:** 2024-12-18

**Authors:** Ana Rita Matias

**Affiliations:** 1 University of Évora, Evora, Portugal

Dear Authors,

 Thank you for submitting to the F1000Research such an interesting manuscript.

 Generally, some concepts need to be clarified, and also some English parts should be reviewed.

 After a careful reading, I have a few questions and suggestions, which I hope will help to improve your manuscript.

**ABSTRACT**

 BACKGROUND

- I suggest rewriting the following paragraph: “In turn, teaching methodologies influence motor learning, as well as psychological, cognitive and social learning, with different impacts on human development and learning retention, levels of intrinsic motivation and continuity of practice in order to support a healthy lifestyle.”
- Suggestion: Teaching methodologies influence learning, human development, motivation levels and, consequently, continuity of practice in favour of a healthy lifestyle.
- “… aquatic literacy is an integral part of physical literacy and the only possibility of being more able to interact with this environment.? – This sentence's meaning is unclear.
- At the end of this section, the two main objectives should be pointed out: 1. Assess the professionals' self-efficacy and 2) identify the methodology used by teachers who perceive themselves as most effective. These objectives should be aligned with the research questions on page 5 (5 ^th^ paragraph).

METHODS

 MAC abbreviation should be introduced the first time it is mentioned.

 CONCLUSIONS

 The abstract should be aligned with the background, methods, and conclusions.

**INTRODUCTION**

 P.3 “Healthy variability in teaching” does not clearly explain the meaning of this concept. It probably could be replaced by “There are different approaches regarding the interaction between teacher and student…”

 P.4

 3 ^rd^ paragraph

 “athletes” - It is suggested to use the same word throughout the text: student, subjects, athletes…

 6 ^th^ paragraph

 “…especially considering that many students would opt out of physical education if given the choice, with over 50% of 10th and 11 ^th^ graders expressing disinterest in the current curriculum” - This sentence's meaning is partly unclear.

 “…used by the teacher can help participants to achieve their present and subsequent achievements.” – This sentence's meaning is partly unclear.

 P.5

 6 ^th^ paragraph

 This paragraph should be rewritten to keep the goal of this study simple.

**MEASURES**

 The authors should mention in which county the questionnaires were administrated and whether they needed to be translated.

 Teaching-Learning Methods Scale in Physical Education: The authors mention five dimensions but only present four.

**DISCUSSION**

 It seems that all statistical procedures were correctly chosen.

 _________________

**FINAL REMARKS**

 Despite being an article with a pertinent theme for the field, it needs some improvement.

 Apart from the above-mentioned suggestions, I suggest removing the data relating to the application of the MAC instrument.

 This study should focus on two objectives:

 1) assessing teachers' perceived self-efficacy by applying the Teachers’ Sense of Efficacy Scale and

 2) identifying which methodology is used by teachers who perceive themselves to be more effective, using the TLMS-PE.

 MAC’s data should be integrated into a second article.

Is the work clearly and accurately presented and does it cite the current literature?

Partly

If applicable, is the statistical analysis and its interpretation appropriate?

Partly

Are all the source data underlying the results available to ensure full reproducibility?

Partly

Is the study design appropriate and is the work technically sound?

Partly

Are the conclusions drawn adequately supported by the results?

Yes

Are sufficient details of methods and analysis provided to allow replication by others?

Partly

Reviewer Expertise:

Aquatic therapy; child development; learning development

I confirm that I have read this submission and believe that I have an appropriate level of expertise to confirm that it is of an acceptable scientific standard, however I have significant reservations, as outlined above.

# Response

**Published:** 2025-02-14

**Authors:** Rita Pinto

**Affiliations:** Universidad Miguel Hernandez de Elche, Elche, Valencian Community, Spain

Thank you for your valuable comments and references to adjust the document to improve clarity and value. We hope that we attend positively to all your questions. We present the answer respecting the order of your comments.

 - **Rewriting paragraphs**

 We have reviewed and modified the paragraph according to your recommendations. The revised text now reads as follows: "Teaching methodologies influence learning, human development, motivation levels, and consequently, the continuity of practice in favour of a healthy lifestyle."

 We have taken your suggestion into account and have rewritten the paragraph as proposed. The text now reads as follows:

 "Teaching methodologies influence learning, human development, motivation levels, and consequently, the continuity of practice in favour of a healthy lifestyle."

 We have rewritten the sentence to clarify its meaning. The revised text now reads as follows: "Water literacy is an integral part of physical literacy and essential for interacting safely and effectively with the aquatic environment."

 - The two main objectives should be pointed out: 1.  

 We have rewritten the text, adjusting the methods to make the objectives clearer. At the end of this section, we have explicitly highlighted the two main objectives:

 To evaluate the self-efficacy of professionals.

 To identify the methodology used by teachers perceived as more effective.

 Additionally, we have reviewed these objectives to ensure they align with the research questions on page 5 (5th paragraph) to maintain coherence throughout the manuscript.

 - MAC abbreviation should be introduced the first time it is mentioned.

 Thank you for pointing that out. We have corrected the manuscript by introducing the abbreviation MAC the first time it is mentioned, as indicated.

 - **Introduction**

 P3 - Thank you for your suggestion. We have rewritten the paragraph as indicated.

 P4 (3th and 6th paragraph)- Thank you for your suggestion. We have rewritten the paragraph as indicated.

 P5 - We have rewritten the paragraph as indicated use as reference the objectives of the study mentioned on abstract.

 - **Methods**: The authors should mention in which country the questionnaires were administrated and whether they needed to be translated

 We have rewritten the Measures section to clarify that the three questionnaires used were adapted into a single one, so that participants cannot identify which content they are responding to. Additionally, we have specified that the translation was done into Spanish, Portuguese, and Brazilian Portuguese.

 We have also included information about the countries where the questionnaires were administered, ensuring that the translations were necessary to guarantee proper understanding by the participants.

**Discussion**

 Thank you for your valuable comment. We understand the concern raised regarding the lack of clarity in the conceptual distinction between the Comprehensive Aquatic Method (MAC) and teaching methodologies in general, especially in relation to its comparison with constructivist methodologies.

 We would like to clarify that, although the MAC is described as a constructivist approach, it is not solely a constructivist methodology in the strict sense. The MAC is an integrative approach that combines various learning theories, such as constructivism, cognitivism, and student-centered elements. While it is based on the constructivist principles that emphasize active participation and adaptation to the individual needs of students, the MAC also includes strategies and structures from other pedagogical approaches aimed at fostering more comprehensive learning tailored to the context of aquatic education.

 This broader approach of the MAC could explain the observed results, which report that this methodology promotes greater self-efficacy in teachers compared to purely constructivist methodologies. By integrating a variety of strategies that go beyond what is traditionally associated with constructivism, such as structured support and learning organization, the MAC allows teachers to feel more confident and competent in their practice.

 We have reviewed the manuscript and believe it is important to clarify this point. Rather than referring to the MAC solely as a "constructivist methodology," we propose describing it as an integrative pedagogical approach that uses various learning theories to create a more dynamic and effective teaching environment for both students and teachers.

**Conclusions**

 The conclusions have been aligned with the background, methods, and research objectives.

 ----

 Thank you for your suggestions and comments. We would like to clarify that, for us, it is essential to understand how the Comprehensive Aquatic Method (MAC) relates to teacher self-efficacy and the methodologies used in the educational field. The MAC is not only a pedagogical approach, but we also believe it directly influences teacher self-efficacy, making the inclusion of this instrument in the study relevant to analyse this relationship.

 While we recognize the importance of focusing on the two proposed objectives—evaluating perceived self-efficacy through the Teacher Sense of Efficacy Scale and identifying which methodology is used by the most effective teachers through the TLMS-PE we believe that the data related to the application of the MAC adds value to the study. This data provides a more comprehensive context on how methodologies influence teachers' self-efficacy perceptions, particularly how the MAC could enhance it in comparison to other methodologies.

# Reviewer report: Reviewer response for version 1

**DOI:** 10.5256/f1000research.165114.r341212

**Published:** 2024-12-14

**Authors:** Carolina Burnay

**Affiliations:** 1 Universidade de Lisboa, Lisbon, Lisbon, Portugal

The article investigates the relationship between teaching methodologies and self-efficacy in aquatic educators. Key findings include:

- Constructivist and cognitive approaches, which emphasize active student participation, are most effective in enhancing teacher self-efficacy.
- The Comprehensive Aquatic Method (MAC), rooted in constructivist approach, is also positively linked to teacher self-efficacy.
- Behaviorist approach and laissez-faire methodologies are less effective in building teacher self-efficacy.

Based on responses from 607 aquatic educators, the authors report that active, student-centered methodologies positively correlated with teacher self-efficacy, while passive approaches showed weaker associations.

 The study advocates for adopting cognitive and constructivist teaching approaches, more specifically, the MAC methodology, in aquatic education to improve teacher confidence and encourage healthier, more active lifestyles among students.

 However, some areas would benefit from further explanation or clarification.

 1. The authors reference developmental and learning theories such as constructivism, cognitivism, and behaviorism, treating them as though they were teaching methodologies. Teaching methodologies, like the cited MAC, can be developed using strategies underpinned by these theories. However, it is important to note that constructivism, cognitivism, and behaviorism are theoretical frameworks, not methodologies. By conflating these theories with methodologies, the authors claim to be investigating which methodology promotes higher levels of self-efficacy in teachers. This distinction between theories and methodologies should be clarified to ensure accuracy and alignment with the study's objectives.

 2. It is unclear which methodologies the authors are analyzing in relation to teachers' self-efficacy. In the Introduction (Teaching Methodologies) and Methodologies sections, the authors refer to:

- Reproductive methodology – teacher direct approach
- Productive/learner centered methodology – problem solving, underpinned by the constructivist theory
- Constructivist methodology - active participation, enabling students to solve problems with teacher support
- Laissez-faire - teacher only organizes equipment and manages time and space without engaging with students during activities

But in the last paragraph of Introduction and in the Results sessions, when stating the main goals of the study, the authors refer to:

- cognitive,
- constructivist,
- behaviourist
- laissez-faire

To improve clarity and consistency, it is crucial to explicitly define the variables under analysis and use the same terms consistently throughout the manuscript. This alignment will help avoid confusion and ensure the study's findings are clearly communicated.

 3. While the Comprehensive Aquatic Method (MAC) is highlighted as effective, the manuscript provides limited quantitative comparisons between MAC and other methodologies. The authors describe the MAC as “a constructivist approach that centers on student interests and needs.” However, the results suggest that MAC methodology promotes higher self-efficacy in teachers than constructivist methodologies. This creates confusion regarding the definition and distinction of teaching methodologies, which requires clearer explanation and better conceptual alignment.

 4. In the Methods/Measures section, the authors mention that three questionnaires were used, but only one is available online. Additionally, in the last sentence of the Procedures section, the authors refer to “the questionnaire,” making it unclear whether participants were asked to complete three separate questionnaires or a single questionnaire that combines questions from the three mentioned in the Methods/Measures section. This ambiguity needs clarification to ensure the methodology is fully understood.

 Therefore, while the study provides valuable insights into the relationship between teaching methodologies and self-efficacy in aquatic education, addressing the identified issues of conceptual clarity, methodological consistency, and writing quality would significantly enhance the manuscript's overall impact and readability.

Is the work clearly and accurately presented and does it cite the current literature?

Partly

If applicable, is the statistical analysis and its interpretation appropriate?

Partly

Are all the source data underlying the results available to ensure full reproducibility?

Yes

Is the study design appropriate and is the work technically sound?

Partly

Are the conclusions drawn adequately supported by the results?

No

Are sufficient details of methods and analysis provided to allow replication by others?

No

Reviewer Expertise:

Child Motor Development.

I confirm that I have read this submission and believe that I have an appropriate level of expertise to confirm that it is of an acceptable scientific standard, however I have significant reservations, as outlined above.

# Response

**Published:** 2025-02-14

**Authors:** Rita Pinto

**Affiliations:** Universidad Miguel Hernandez de Elche, Elche, Valencian Community, Spain

We appreciate your comments and have reviewed the entire document to align it with your suggestions. We will present our response, respecting the order of the comments made. We hope to respond positively to all requests.

 - In particular, we have revised the relevant sections to clarify the distinction between learning theories and teaching methodologies.

 - Additionally, we have clarified that the study aims to analyze which teaching methodology, grounded in these theories, fosters higher levels of self-efficacy in teachers. This distinction is crucial to ensuring consistency with the research objectives.

 - In particular, we have standardized the terminology used to describe the methodologies analyzed in relation to teacher self-efficacy, ensuring consistency across the Introduction, Methodology section, and Results. We have clarified the definition of each methodology under analysis and ensured the consistent use of terms throughout the manuscript. This helps to avoid potential confusion and enhances the clarity of the study’s findings. We this adjustments to the manuscript we believe that we improved clarity and conceptual consistency in the definition and distinction of teaching methodologies. Thank you for your comments.

 -We have revised the description of the Comprehensive Aquatic Method (MAC) to clarify its theoretical foundation and its relationship to constructivism. While MAC incorporates constructivist principles, it also integrates strategies from other approaches, which may explain why the results show higher teacher self-efficacy compared to purely constructivist methodologies.

 Additionally, we have included a more detailed explanation of the differences between MAC and other methodologies, as well as a clearer analysis of the factors that might influence teacher self-efficacy levels. We have also strengthened the discussion on the need for further comparative studies that provide additional quantitative evidence.

 - We appreciate your comment and have made the necessary modifications in the Methods/Measures section to clarify the identified ambiguity. We have rewritten the description of the measures used, explaining that, although three questionnaires were initially mentioned, they were adapted and combined into a single questionnaire. This clarification eliminates any confusion regarding whether participants completed three separate questionnaires or just one. Additionally, we have adjusted the final sentence of the Procedures section to accurately reflect that a single questionnaire was used, incorporating questions from the three original questionnaires.

 - The Physical Education Teaching-Learning Methods Scale has four dimensions, not five as previously mentioned. We have corrected this inconsistency in the manuscript, and it now correctly describes that the scale consists of four dimensions.

 Thank you very much.

# Reviewer report: Reviewer response for version 1

**DOI:** 10.5256/f1000research.165114.r341216

**Published:** 2024-12-09

**Authors:** Qi Tian

**Affiliations:** 1 Jingchu University of Technology, Jingmen City, China

The paper explores the relationship between teachers' self-efficacy and teaching methods through an extensive review of the literature. The conclusions drawn from the study are credible and provide valuable insights for enhancing both teachers’self-efficacy and their teaching practices. However, the article has two notable issues that need to be addressed:

 firstly, **Insufficient Recent Literature**: The paper cites a limited amount of literature from the past five years, comprising only 17.2% (15 out of 87) of the total references. This raises concerns about the relevance of the supporting evidence to current teaching contexts.

 Secondly, **Statistical Scoring Conversion**: The teacher self-efficacy scale differs from the other two scales in that it is not based on a 5-point scale. The article does not provide a clear explanation of how the scores from this scale were converted for statistical analysis, which could affect the reliability of the findings. To address these concerns, it is recommended that the authors update their references to include more recent studies, particularly from the last five years, to ensure the literature review reflects current trends and developments. Additionally, a detailed description of the methodology used for converting the teacher self-efficacy scores into a format compatible with statistical analysis should be included. These revisions will enhance the article's rigor and comprehensiveness.

**Suggestion**: With these improvements, the article can be revised and considered for approval.

Is the work clearly and accurately presented and does it cite the current literature?

Partly

If applicable, is the statistical analysis and its interpretation appropriate?

Partly

Are all the source data underlying the results available to ensure full reproducibility?

Yes

Is the study design appropriate and is the work technically sound?

Yes

Are the conclusions drawn adequately supported by the results?

Yes

Are sufficient details of methods and analysis provided to allow replication by others?

Yes

Reviewer Expertise:

Educational psychology

I confirm that I have read this submission and believe that I have an appropriate level of expertise to confirm that it is of an acceptable scientific standard, however I have significant reservations, as outlined above.

# Response

**Published:** 2025-02-14

**Authors:** Rita Pinto

**Affiliations:** Universidad Miguel Hernandez de Elche, Elche, Valencian Community, Spain

We appreciate your valuable comments on our article. We will present our response, respecting the order of the comments made. We hope to respond positively to all requests.

 - New references from the last five years have been included: Luo et al., 2024; Hussain & Khan, 2022; Wettstein et al., 2021; Li, 2023; Salas-Rodríguez et al., 2021; Barni et al., 2019; Lauermann & ten Hagen, 2021.

 - We would like to address your concern regarding the teacher self-efficacy scale and the conversion of its scores for statistical analysis. First, it is important to highlight that the teacher self-efficacy scale used in our study follows a methodological approach that has been widely validated in the literature, allowing for comparison with other scales even when they use a different metric. The decision to employ a different scale is based on the original structure of the instrument, whose validity and reliability have been demonstrated in previous studies.

 We do not consider normalization or standardization of the data necessary, as comparisons can be made directly without compromising the validity of the results. Differences in scale metrics do not affect the analysis, as comparisons focus on score trends rather than absolute values. This approach is widely accepted in studies of this nature and has been supported by methodological literature.

 Thank you very much
